# Paradoxic effects of propofol on visceral pain induced by various TRPV1 agonists

**DOI:** 10.3892/etm.2013.950

**Published:** 2013-02-05

**Authors:** WENJIN JI, CAN CUI, ZHIWEI ZHANG, JIEXIAN LIANG

**Affiliations:** 1 Postgraduate Institute, Southern Medical University, Guangzhou 510015;; 2 Departments of Anesthesiology, Guangdong Cardiovascular Institute, Guangdong General Hospital, Guangdong Academy of Medical Sciences, Guangzhou 510080, P.R. China; 3 Pediatric Cardiology, Guangdong Cardiovascular Institute, Guangdong General Hospital, Guangdong Academy of Medical Sciences, Guangzhou 510080, P.R. China

**Keywords:** propofol, visceral pain, acetic acid, capsaicin, transient receptor potential vanilloid subtype-1

## Abstract

Intraperitoneal injection of propofol inhibits subsequent acetic acid-induced writhing response in mice. Propofol increases the sensitivity of dorsal root ganglion neurons to capsaicin through transient receptor potential ankyrin subtype-1 (TRPA1) and protein kinase Cε (PKCε)-mediated phosphorylation of transient receptor potential vanilloid subtype-1 (TRPV1). Intraperitoneal co-injection of propofol may increase visceral nociception induced by TRPV1 agonists via sensitization of TRPV1. Therefore, we investigated the effects of intraperitoneal co-injection of propofol on nociception induced by acetic acid and capsaicin. The number of writhing movements induced by acetic acid or nociception time by capsaicin with or without propofol were counted. Neonatal capsaicin-treated mice were also used to demonstrate the role of TRPV1 in the effects of propofol on nociception, induced by TRPV1 agonists. Co-injection of propofol resulted in a pronociceptive effect on the writhing response induced by acetic acid, while the same dose of propofol ameliorated the response to capsaicin. The writhing response to intraperitoneal acetic acid was sharply inhibited following neonatal treatment with capsaicin. Co-injection with propofol reduced the number of writhing movements induced by acetic acid in neonatal capsaicin-treated mice. These results suggest that propofol binds to TRPV1 at the capsaicin-binding pocket.

## Introduction

Propofol is a widely used general anesthetic with a high incidence of injection pain when administered intravenously (1). However, the anti-nociceptive properties of propofol have been demonstrated in several studies (2–6). A previous study demonstrated that propofol decreases nerve excitability of primary sensory afferents (7). Local injection of propofol produces a dose-dependent anti-nociceptive effect on the early and late phases of the formalin test (2). Another study identified that co-injection of propofol inhibits the pain behavior evoked by bee venom (6). Intraperitoneal injection of propofol in mice inhibits the acetic acid-induced writhing response through a spinal mechanism (4).

The capsaicin receptor, transient receptor potential vanilloid subtype-1 (TRPV1) plays an important role in pain signaling and is essential for the development of inflammatory thermal hyperalgesia in mice (8–10). Activation of TRPV1 is vital in visceral pain. TRPV1 antagonists or depletion of TRPV1 neurons by neonatal capsaicin injection inhibit writhing caused by acetic acid (11–13). A series of factors, including endothelin-1, prostaglandins and bradykinin, sensitize the response of TRPV1 mainly through the protein kinase A (PKA) or protein kinase C (PKC) pathways to phosphorylate TRPV1 (14–16). Propofol increases the sensitivity of dorsal root ganglion neurons to capsaicin through transient receptor potential ankyrin receptor subtype-1 (TRPA1) and PKCε-mediated phosphorylation of TRPV1 (17,18). Intraperitoneal co-injection of propofol may increase visceral nociception induced by TRPV1 agonists via sensitization of TRPV1, which is paradoxic to the anti-nociceptive effects of propofol. Therefore, we investigated the effects of intraperitoneal co-injection of propofol on noceception induced by acetic acid and capsaicin.

## Materials and methods

### Animals

Male C57BL/6J mice, weighing 20–22 g, were purchased from the Center for Laboratory Animals, Sun Yat-Shen University (Guangzhou, China). The mice were housed at room temperature (22±1°C) on a 12/12-h light (8am–8pm)/dark (8pm–8am) cycle and had free access to rodent chow and water. The experimental procedures and the animal use and care protocols were approved by the Committee on Ethical Use of Animals of Guangdong General Hospital (Guangzhou, China). The procedures also followed the animal use and care guidelines of the National Institutes of Health. All efforts were made to reduce the number of animals used and to minimize animal suffering.

### Drugs and chemicals

Propofol (2,6-diisopropylphenol) and capsaicin were purchased from Sigma (St. Louis, MO, USA) and dissolved in dimethylsulfoxide (DMSO) as stock solution. Ice acetic acid was purchased from Hangzhou Chemical Reagents Co., Ltd. (Zhejiang, China). All drugs were diluted in normal saline prior to use.

### Nociception induced by acetic acid and capsaicin

Mice were acclimatized to the testing environment (clear Plexiglas box) for 30 min. Care was taken while handling the animals in order to minimize stress. Mice were injected intraperitoneally using a 27 gauge needle, in the left lower quadrant of the abdomen with 0.01 ml/g body weight of a 0.6% acetic acid solution plus 5 mM propofol or 200 μM capsaicin plus 5 mM propofol (n=10 for each group). The final concentration of DMSO in the solution for intraperitoneal injection was adjusted to 2.5% for acetic acid or 4.5% for capsaicin. Following injection, the animals were returned to the chamber and their subsequent nociceptive behavior observed. The number of writhing movements (‘writhing’ consists of contractions of the abdomen, twisting and turning of the trunk, arching of the back and extension of the hind limbs) were counted for 30 min after injection of acetic acid. The amount of time spent recumbent (either lying on the stomach or sitting in a hunched position with head down, not moving) was recorded for 10 min after injection of capsaicin (19).

### Capsaicin treatment in neonatal mice

To demonstrate the role of TRPV1 on the effects of propofol, male neonatal mice (aged 2 days) were anesthetized with sevoflurane and injected subcutaneously with capsaicin, a TRPV1-depleting agent (50 mg/kg) or the vehicle [10% ethanol, 10% Tween-80 and 80% phosphate-buffered saline (PBS)] as described previously (20). Animals were included in the study 6 weeks after the administration of capsaicin or the vehicle. The effects of capsaicin were expected to cause depletion of TRPV1 neurons and was verified by the eye-wiping test. For this test, capsaicin (0.01%, 20 μl) was sprayed into the eye and the number of wiping movements that occurred within 1 min was counted. The animal was considered to be desensitized to TRPV1 by neonatal capsaicin treatment when the animal wiped its eyes no more than five times.

### Statistical analysis

Minitab 16 for Windows (Minitab Inc, PA, USA) was used to carry out statistical analyses. All data are presented as mean ± standard deviation (SD). Data were statistically evaluated by analysis of variance followed by Bonferroni’s test. P<0.05 was considered to indicate a statistically significant difference.

## Results

### Intraperitoneal injection of acetic acid

**Figure 1:**
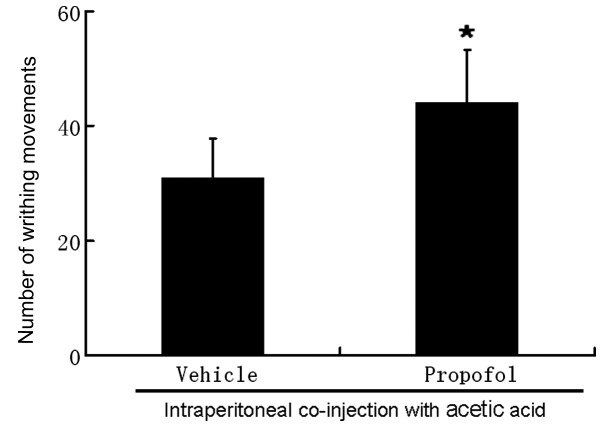
Effects of co-adminstration of propofol on the writhing response induced by intraperitoneal injection of acetic acid. A total volume of 0.01 ml/g body weight of a 0.6% acetic acid solution with 5 mM propofol (propofol group) or without propofol (vehicle group) was injected intraperitoneally. The final concentration of dimethylsulfoxide (DMSO) in the solution for intraperitoneal injection was adjusted to 2.5%. Each bar indicates the number of writhing movements in the 30 min after injection of acetic acid. Data are expressed as mean ± standard deviation (SD); n=10 in each treatment group. ^*^P<0.05, compared to the group treated with acetic acid only.

Intraperitoneal injection of propofol at 5 mM did not cause pain-related behavior in mice. Intraperitoneal injection of 0.6% acetic acid evoked an average of 31±6.9 writhing responses with a latency <2 min. We counted the number of writhing movements for 30 min as the majority of the writhing responses occurred within 30 min of injection with acetic acid. Co-administration of propofol 5 mM with acetic acid increased the number of writhing movements to 42.8±9.2 (P<0.05, Fig. 1).

### Intraperitoneal injection of capsaicin

**Figure 2:**
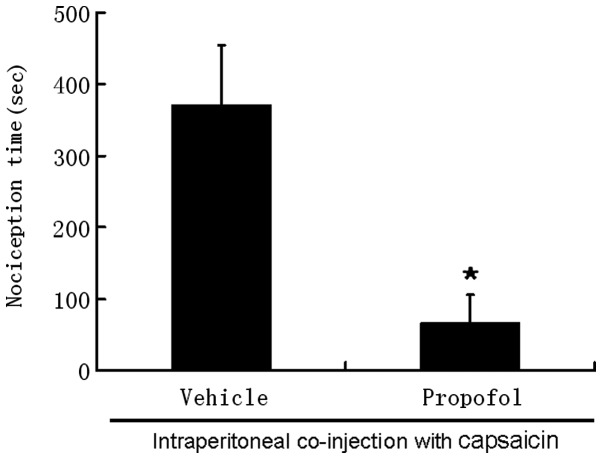
Effects of co-adminstration of propofol on the nociception time induced by intraperitoneal injection of capsaicin. A total volume of 0.01 ml/g body weight of a 200 μM capsaicin solution with 5 mM propofol (propofol group) or without propofol (vehicle group) was injected intraperitoneally. The final concentration of dimethylsulfoxide (DMSO) in the solution for intraperitoneal injection was adjusted to 4.5%. Each bar indicates the amount of time spent recumbent in a 10 min period following the injection of capsaicin. Data are expressed as mean ± standard deviation (SD); n=10 in each treatment group. ^*^P<0.05, compared to the group treated with capsaicin only.

Intraperitoneal injection of 200 μM capsaicin evoked pronounced nociceptive behaviors within 10 sec of injection. These behaviors disappeared 10 min after injection. The average nociception time induced by 200 μM capsaicin was 371±82.7 sec. The nociception time was sharply reduced to 67±39 sec following co-administration of capsaicin with propofol (P<0.05; Fig. 2).

### Contribution of C-fiber activation

**Figure 3:**
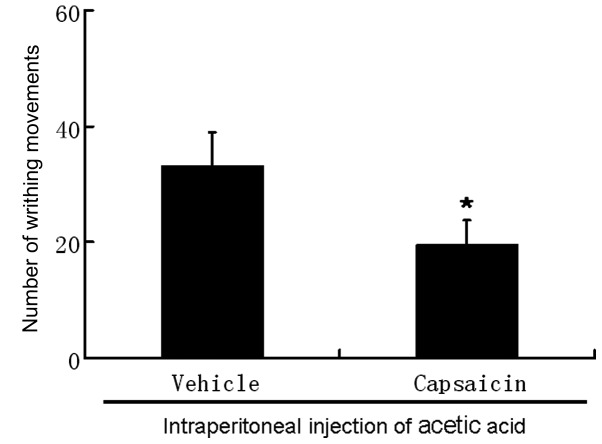
Effects of neonatal capsaicin treatment on the writhing response induced by intraperitoneal injection of acetic acid. A total volume of 0.01 ml/g body weight of a 0.6% acetic acid solution was injected intraperitoneally. Each bar indicates the number of writhing movements in the 30 min after injection of acetic acid. Data are expressed as mean ± standard deviation (SD); n=10 in each treatment group. ^*^P<0.05, compared to the group treated with vehicle only in the neonatal period.

**Figure 4:**
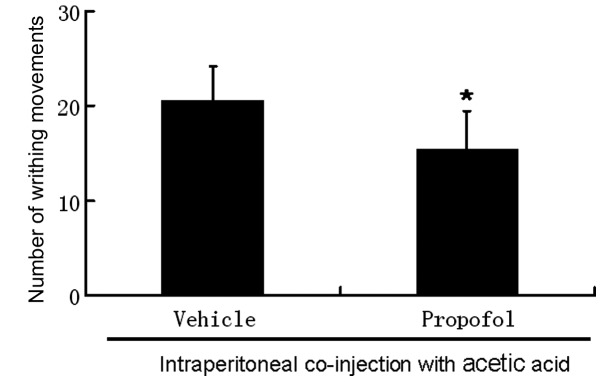
Effects of co-adminstration of propofol on the writhing response induced by intraperitoneal injection of acetic acid in neonatal capsaicin-treated mice. A total volume of 0.01 ml/g body weight of a 0.6% acetic acid solution with 5 mM propofol (propofol group) or without propofol (vehicle group) was injected intraperitoneally. The final concentration of dimethylsulfoxide (DMSO) in the solution for intraperitoneal injection was adjusted to 2.5%. Each bar indicates the number of writhing movements in the 30 min after injection of acetic acid. Data are expressed as mean ± standard deviation (SD); n=10 in each treatment group. ^*^P<0.05, compared to the group treated with acetic acid only.

To determine the contribution of C-fiber activation to the effects of propofol in the algogens-induced nociceptive behavior in mice, neonatal animals (aged 2 days) were subcutaneously treated with capsaicin (50 mg/kg) and underwent acetic acid or capsaicin-induced nociception when they became adults. Mice treated with capsaicin did not spend any time recumbent following intraperitoneal injection of capsaicin and demonstrated a marked reduction in the number of writhing movements (20±4.1) compared with vehicle-treated animals (33±5.8; Fig. 3) following intraperitoneal injection of acetic acid. Co-administration of propofol reduced the number of writhing movements induced by acetic acid to 16±3.9 compared to that without propofol (21±3.6; Fig. 4) in capsaicin-treated mice.

## Discussion

In this study, co-injection of propofol had a pro-nociceptive effect on the writhing response induced by acetic acid. The writhing response to intraperitoneal acetic acid is mainly mediated by TRPV1 and was sharply inhibited following neonatal treatment with capsaicin in this study and in previous research (13). TRPV1 is a non-selective cation channel with a preference for calcium that is directly activated by capsaicin, protons, cations, noxious temperatures and other endogenous ligands, including lipid metabolites (21). Various agents, including endothelin-1 and bradykinin in the ‘inflammatory soup’, act together to lower the activation threshold of TRPV1 (14–16). Propofol often induces pain when injected into peripheral small veins. Bradykinin and prostaglandins are involved in the pain following injection of propofol (22,23). Propofol restores the sensitivity of TRPV1 receptors following agonist-induced desensitization and attenuates agonist-induced desensitization via PKCε and the TRPA1-dependent pathway in mouse dorsal root ganglion (DRG) sensory neurons (17,18). Acetic acid-induced visceral nociception is reduced by ∼70% in knockout mice lacking B1 and B2 receptors (24). Propofol may increase the nociceptive response to acetic acid through sensitization of TRPV1 or kinin receptors. However, it is hard to interpret the same dose of propofol ameliorating the response to capsaicin.

Several studies reported that the systemic administration of a subhypnotic dosage of propofol produces hyperalgesic effects (25–27) and a large dosage, which results in a loss of the right reflex, produces analgesic effects (25). Intraperitoneal administration of a subhypnotic dose of propofol or microinjection of propofol into the lateral ventricle or ventrolateral periaqueductal gray matter produces significant hyperalgesia assessed by the hot-plate test and formalin test in conscious rats (25), while intrathecal injection of propofol demonstrated an analgesic effect (25). It was concluded that propofol induces hyperalgesia through a superspinal mechanism. Intraperitoneal injection of propofol in mice results in anti-nociceptive effects in the hot-plate test and acetic acid induces the writhing response through a spinal *N*-methyl-D-aspartic acid (NMDA) and α-amino-3-hydroxy-5-methyl-4-isoxazolepropionic acid (AMPA) mechanism (4). Co-injection of propofol inhibits the pain behavior evoked by bee venom (6). Generally speaking, the dosage and route of administration determine the hyperalgesic or analgesic effect of propofol. In contrast to previous reports, we identified that co-injection of propofol exerts hyperalgesic and anti-nociceptive effects. It increases the number of writhing movements induced by acetic acid and decreases the nociception time evoked by capsaicin. Our results suggest that TRPV1 is involved in the effect of propofol in inhibiting the writhing response evoked by acetic acid in neonatal capsaicin-treated animals.

TRPA1 mediates propofol-induced pain behavior caused by intranasal propofol and flexor reflex response following intra-arterial propofol (23,28). Propofol evokes an inward current only in TRPA1-expressing neurons (28,29) and fails to evoke this current in HEK293 cells transfected with TRPV1 (28). Propofol does not alter the response of recombinant rat TRPV1 to capsaicin (30). However, there is conflicting evidence on the alteration of TRPV1 by propofol. A previous study identified that propofol increases the intracellular calcium concentration in HEK 293 cells transfected with Wistar rat TRPV1 cDNA (31). Co-application of capsaicin and propofol results in even smaller currents than when capsaicin is applied alone in HEK 293 cells transfected with human TRPV1 cDNA (32). These studies suggest that propofol has a functional interaction with TRPV1.

Through direct binding to TRPV1, certain agonists also act as potentiation mediators; for example, mild acidification will lower the temperature threshold and enlarge the heat- or capsaicin-induced current (33,34). Chick TRPV1 is not sensitive to capsaicin; however, high concentrations of capsaicin enhance the proton-evoked current (35). When the binding site is overlapped, the response is inhibited due to competitive binding, as cations act at TRPV1 proton binding residues E600 and E648 (34,36). Divalent cations, including Mg, sensitize TRPV1 at 1–10 mM Mg and block the response to protons at 5–10 mM Mg (36). Propofol possibly acts on the receptor inside the cell membrane or hydrophobic regions of the receptor penetrating the lipid bilayers, since propofol is lipophilic and permeable to the cell membrane (37,38). Propofol enhanced the writhing response to acetic acid and reduced the nociceptive response to capsaicin in the current study, which suggests that propofol binds to TRPV1 at the capsaicin binding site, which is a hydrophobic region of the receptor.

Propofol is a widely used general anesthetic and the mechanism of general anesthesia induced by propofol is still under investigation. In the current study, *in vivo* results suggest that propofol binds to TRPV1 at the site of the capsaicin binding pocket. Further investigation is required to explore how propofol binds to ion channels.
